# RNA-Based COVID-19 vaccine candidates with clinical phase trials in progress

**DOI:** 10.3906/sag-2104-139

**Published:** 2021-12-17

**Authors:** Leyla İpek RUDVAN AL, Meliha Çağla SÖNMEZER, Serhat ÜNAL

**Affiliations:** 1 Department of Infectious Disease and Clinical Microbiology, Faculty of Medicine, Hacettepe University, Ankara Turkey

**Keywords:** SARS-CoV2, COVID-19, vaccine trials, m-RNA

## Abstract

Due to the COVID-19 infection, which was recognized as a global pandemic by the WHO on March 11, 2020, the number of cases and disease-related deaths increases day by day globally. For this reason, antiviral agents used in treatment and vaccines, the most effective weapon in prevention, continue to be the most popular topic of the plan. Several situations are expected to affect the course of the pandemic. The loss of the ability of the virus to mutate and cause disease, the fact that those who become immunized by having the disease in the society reach a critical rate and create social immunity (herd immunity), and the provision of social immunity with effective vaccination can be counted as some of these situations.Candidate vaccines in the clinical phase among RNA-based vaccines: This review aimed to examine COVID-19 vaccine candidates using RNA technology and compile its current data. We used PubMed, Google Scholar, and World Health Organization (WHO) databases. Also, we followed up on the latest news and developments on vaccine companies’ websites.Conclusion: Vaccination trials, which started due to the seriousness and urgency of the situation that we are in, continue exceptionally quickly and effectively. As per the WHO›s data on July 9, 2021, there have been 291 vaccine trials, 107 of which are in the clinical phase, and 18 (16%) of the vaccine candidates in the clinical phase are RNA-based vaccines. Also, the number of RNA-based vaccines with ongoing preclinical trials is 2

## 1. Introduction 

With the investigation of pneumonia cases, which first appeared in Wuhan, China, in December 2019, seen in different cities outside Wuhan in January 2020, the actual cause could not be fully revealed. It was revealed that the factor was a new type of coronavirus on January 7, 2020 [1]. This virus has been named 2019-nCoV (2019-novel coronavirus) by the World Health Organization (WHO) and as SARS-CoV-2 (severe acute respiratory syndrome coronavirus-2) by the International Virus Taxonomy Committee. International Committee on Taxonomy of Viruses (2021) [online] Website https://talk. ictvonline.org [accessed 20 January 2021]. Also, the disease caused by the virus is named COVID-19 (coronavirus disease-2019). The disease typically progresses with respiratory system symptoms, but it can also progress multisystemic due to the endothelial damage it causes. Cardiovascular and central nervous system involvement, bleeding diathesis, and ocular findings are frequently observed [2–6]. 

WHO declared COVID-19 as a global pandemic on March 11, 2020. World Health Organization (2020) [online] Website https://www.who.int/director-general/speeches/detail/who-director-general-s-opening-remarks-at-the-media-briefing-on-covid-19 [accessed 11 March 2020]. According to WHO data, the total number of confirmed COVID-19 cases worldwide as of July 9, 2021 is 185,291,530, and the total number of reported deaths due to disease has reached 4,010,834. The first COVID-19 case in Turkey was seen on March 10, 2020, and the first death reported due to this disease was on April 11, 2020. When countries worldwide are ranked according to the total number of COVID-19 cases, the United States of America (USA) comes first, followed by the India-Brazil-Russia-France. Our country is in fifth place in this ranking with a total number of 5,465,094 cases. World Health Organization Coronavirus Disease (COVID-19) Dashboard (2021) [online] Website https://covid19.who.int/ [accessed 20 April 2021]. As in our country, the number of people infected with COVID-19 and deaths due to disease increases day by day. Republic of Turkey Ministry of Health General Directorate of Public Health (2021) [online] Website https://hsgm.saglik.gov.tr/tr/covid19 [accessed 11 March 2021]. Because of this situation, both vaccines and antiviral agents used in treatment gain more importance, and accordingly, they occupy the agenda intensely. 

As with many infectious diseases, vaccines are critical in preventing COVID-19 infection. According to the WHO’s data, on July 9, 2021, the number of COVID-19 vaccine trials worldwide is 291 in total; 107 of them are in the clinical phase, while 184 are in the preclinical stage. Among the RNA-based vaccines subject to our review, the number of vaccines in the clinical phase is 18, while the number in the preclinical phase is 24. World Health Organization The DRAFT Landscape of COVID-19 Candidate Vaccines (2021) [online] Website https://www.who.int/publications/m/item/draft-landscape-of-covid-19-candidate-vaccines [accessed 11 March 2021].

The distribution of 107 vaccine candidates, of which clinical trials are in progress, as per the technologies they use, is shown in Table 1.

**Table 1:** RNA-based COVID-19 vaccine candidates with used technologies.

Platform	Used technologies	Candidate vaccines (n) (%)
PS	Protein subunit	36 (34%)
VVnr	Viral vector (nonreplicating)	15 (14%)
DNA	DNA	10 (9%)
IV	Inactivated virus	16 (15%)
RNA	RNA	18 (17%)
VVr	Viral vector (replicating)	2 (2%)
VLP	Virus-like particle	5 (5%)
VVr + APC	VVr + Antigen presenting cell	2 (2%)
LAV	Live attenuated virus	2 (2%)
VVnr + APC	VVnr + Antigen presenting cell	1 (1%)
Total		107 (100%)

New vaccine development trials are performed in 4 phases, just like the development of new drugs. In Phase I, vaccines are generally administered to a volunteer population of 50–100 people to determine the safety and effective dose. Vaccines found to be effective and safe in phase I are tested within Phase II trials in larger numbers of healthy volunteers of different ages and sometimes with underlying diseases (usually placebo-controlled). In a classic Phase II trial, the number of volunteers ranges from 400–1000. At this stage, vaccines, which are effective and reliable, are used in Phase III with placebo control in volunteer populations with varying ages and health/underlying disease characteristics, expressed in tens of thousands. Vaccines that are found to be effective and safe due to this phase trials apply to the relevant authorities for authorization approval. Centers for Disease Control and Prevention Vaccine testing and the approval process (2021) [online] Website https://www.cdc.gov/vaccines/basics/test-approve.html [accessed 11 March 2020]. 

The trials conducted after the vaccine is authorized (postmarketing) are called Phase IV trials. Most phase trials were conducted in combination as Phase I and II/Phase II and III during the COVID-19 pandemic. 

In recent years, m-RNA technology has been used in RNA-based vaccines, which have developed rapidly. Many preclinical trials have been found, and the number of clinical trials has increased significantly in the last few years. m RNA is the intermediate step between the translation of protein-encoding DNA and the production of proteins by ribosomes in the cytoplasm. The proteins are transmitted to the ribosomes in the cytoplasm by mRNA transcription, and, thus, the relevant protein synthesis is provided. Naked m-RNA is degraded by extracellular RNases and, therefore, cannot be effectively uptake into the cell. Naked m-RNA is degraded by extracellular RNases and, therefore, cannot be effectively uptake into the cell. This condition disrupts the stability and effectiveness of m-RNA-based vaccines. For this reason, the half-life of mRNAs is extended by ensuring the stability of the 3’ and 5’ UTR regulatory sequences obtained from the viral or eukaryotic genes used. Besides, mRNA stability and protein translation can be achieved by adding the appropriate length of poly-(A)-tail [7]. Various mRNA vaccine platforms have been developed in recent years and validated in studies of immunogenicity and efficacy. Engineering of the RNA sequence has rendered synthetic mRNA more translatable than ever before. Highly efficient and nontoxic RNA carriers have been developed in a way that, in some cases, allows prolonged antigen expression in vivo. Some vaccine formulations contain novel adjuvants, while others elicit potent responses in the absence of known adjuvants. Two major types of RNA are currently studied as vaccines: nonreplicating mRNA and virally derived, self-amplifying RNA. Conventional mRNA-based vaccines encode the antigen of interest and contain 5′ and 3′ untranslated regions (UTRs). In contrast, self-amplifying RNAs encode the antigen and the viral replication machinery that enables intracellular RNA amplification and abundant protein expression.

These vaccines are safe vaccines that do not have an infectious potential, and they cause a potent immune response by stimulating both innate immunity and adaptive immunity with protein translation. Besides, they are stable vaccines with fast-cheap-scalable production potential. RNA-based vaccines generally have to be stored at very low temperatures. This situation emerges as a logistical problem that may be encountered during the vaccination of large human populations. Also, allergic events and autoimmune diseases can be triggered by the potent immune response they cause. 

RNA-based vaccine candidates, of which phase III trials are ongoing, use m-RNA technology encodes the COVID-19 spike protein. Phase III trials have been completed, and the first results are announced in four m-RNA-based vaccines (Pfizer/Biontech-Moderna, CureVac, Academy of Military Science (AMS)-Walvax Biotechnology). Also, two m RNA-based vaccines recently reported the results of Phase IV trials (Pfizer/Biontech and Moderna). Candidate vaccines in the clinical phase among RNA-based vaccines are categorized in Table 2.

**Table 2:** Candidate vaccines in the clinical phase among RNA-based vaccines.

Pfizer-Biontech
Moderna
CureVac
Arcturus Therapeutics
Imperial College London
Academy of Military Science (AMS)- Walvax Biotechnology
Providence Therapeutics
Chulalongkorn University
GlaxoSmithKline
Moderna + National Institute of Allergy and Infectious Diseases (NIAID)
Sanofi Pasteur and Translate Bio.
Daiichi Sankyo Co., Ltd.
ModernaTX, Inc. (m RNA 1273.211 Multivalent Booster )
MRC/UVRI and LSHTM Uganda Research Unit
Shanghai East Hospital
Elixirgen Therapeutics
Senai Cimatec
ModernaTX (m RNA 1283)

### 2.1. Pfizer-Biontech 

On the basis of initial clinical-trial results in Germany, two lipids nanoparticle–formulated, nucleoside-modified RNA (modRNA) vaccine candidates against severe acute respiratory syndrome coronavirus 2 (SARS-CoV-2) were evaluated in the Phase I portion of the trial in the United States. One of these candidates, BNT162b1, encodes the SARS-CoV-2 receptor–binding domain, trimerized by the addition of a T4 fibritin fold on the domain to increase its immunogenicity through the multivalent display. The other candidate, BNT162b2, encodes the SARS-CoV-2 full-length spike, modified by two proline mutations to lock it in the prefusion conformation and more closely mimic the intact virus with which the elicited virus-neutralizing antibodies must interact. Because of the higher reactogenicity of BNT162b1 in older people, BNT162b2 was used in the Phase III trial: the safety, tolerability, and immunogenicity data of Phase I and Phase II trials conducted in Germany. Also, the data were published in October 2020. Accordingly, 60 volunteers were vaccinated with the Pfizer-Biontech vaccine (BNT162b1) between April 23, 2020, and May 22, 2020. The trial was designed with 12 participants in each dose group. Double dose vaccination was made to 1 μg-10 μg-30 μg-, and 50 μg-groups on 1st and 22nd days and single dose to 60 μg-group. Participants consisted of healthy males between the ages of 20–56 and healthy females who were not pregnant, and the median age was 37 years. A total of 96.7% of the participants were Caucasian, 1 participant was African American, and 1 participant was Asian. No severe adverse events were observed in any of the participants (at any dose) followed after vaccination. Systemic side effects reported in the 10 μg to 30 μg groups typically occurred within 24 h following the first injection and are dependent on reactogenicity as expected. Pain and tenderness at the injection site were the most common reactions in the seven days following the 1st and 2nd dose and were reported as dose-dependent and were more common after the 2nd dose. Symptoms such as fever, headache, joint pain, muscle pain, pain, and tenderness at the injection site were often mild/moderate and rarely more severe. However, even severe symptoms are transient and resolved spontaneously or controlled with simple interventions (e.g., paracetamol). Transient CRP elevation and transient decrease in lymphocyte count have been reported after vaccination, both being dose-dependent. The increase in CRP level has been explained as an indicator of the vaccine’s adjuvant molecule activity. Transient decrease in lymphocyte count is related to the redistribution of lymphocytes to lymphoid organs due to stimulation of the innate immune system. Both cases are pharmacodynamic markers that show the activity of RNA vaccines. 

In the trial, baseline RBD-binding Ig G and SARS-CoV-2 neutralizing Ab levels of the participants were examined; control antibody levels were examined 7 and 21 days (days 8–22 and days 29–43) after the 1st and 2nd doses in the other groups, except for the 60 μg group, which was administered as a single dose. Strong vaccine-induced antibody response was reported in a dose-dependent manner in all vaccinated participants. The geometric mean concentration of RBDbinding Ig G (geometric mean concentration/GMC) measured 21 days after the 1st injection in the four dose groups increased dose-dependently (265–1672 unit (U) ml−1). The RBD-binding IgG level in antibody titers measured seven days (29th day) and 21 days (43rd day) after the 2nd dose increased strongly depending on the dose (29. Day GMC: 2015–25.006 U mL−1.) (43. Day GMC: 3.920–18.289 U mL−1 I). 

After the single dose of 60 μg vaccination, the antibody GMC value measured on the 43rd day was reported as 755 U ml−1, and it was stated that a 2nd dose was required to increase the antibody level. 

The SARS-CoV-2 neutralizing antibody geometric mean titer (GMT) measured on the 22nd day following the 1st dose of vaccination was found to be dose-dependent increased. The neutralizing antibody GMT values measured seven days after the second dose were 36 in the 1 μg group, 158 in the 10 μg group, 308 in the 30 μg group, and 578 in the 50 μg group, respectively. Neutralizing antibody GMT value and RBD-binding Ig G GMC titers were found to be decreased 21 days after the 2nd dose of vaccine (43rd day). (Except for 1 μg dose group) Serum virus-neutralizing antibody titer is strongly correlated with RBD-binding IgG titer. 

BNT162b1 results gave similar results with the trial conducted in the USA. 

CD4 (+) and CD8 (+) T cell responses were examined using the ex vivo IFNγ enzyme-linked immunosorbent spot (ELISpot) method on the 1st day before vaccination and seven days (29th day) after the 2nd dose vaccine, and both cell activities are stimulated. As with the antibody titer in the 60 μg single-dose cohort, T cell stimulation was also weaker, reaffirming the importance of the 2nd dose. 

Cytokines were identified to evaluate the function and polarization of RBD-specific T cells stimulated by the vaccine encoded RBD. These cytokines are IFN gamma, IL-2, and IL-4, and they are demonstrated by the intracellular staining method in peripheral mononuclear cells collected before and after vaccination of 52 participants immunized with the vaccine. RBD-specific CD4 (+) T cells secrete IFN gamma, IL-2, or both, but not IL-4. Similarly, CD8 (+) T cells secrete IFN gamma and IL-2. The average fraction of RBD-specific T cells obtained by BNT162b1 vaccination in total circulating T cells was significantly higher than that observed in 15 donors recovering from COVID-19. In summary, these findings suggest that BNT162b1 induces functional and proinflammatory CD4 (+) and CD8 (+) T cell responses in almost all participants with TH1 polarization [8, 9]. 

In December 2020, preliminary data of Phase III trials, in which 43.448 people were randomized in the USA-Argentina-Brazil-Germany-Turkey-South Africa between July 27, 2020, and November 14, 2020, were published. In the trial, 21,720 people were vaccinated with BNT162b2, while 21,728 people formed the placebo arm. A total of 49% of the participants in the trial were women, 83% of white ethnicity, 9% African American, 28% Hispanic (Latin), 35% were obese (people with a body mass index of at least 30), 21% had at least one concomitant medical condition. The participants’ mean age was 52, and 42% of them were over 55 years old. There were 8183 people in the reactogenicity subgroup, most of them were in the vaccine group. The most common local reaction in the vaccine group was mild-moderate pain to the injection site that occurred within seven days after injection. The pain was observed less frequently in individuals over 55 years of age compared to young people. No increase was observed in the local reaction rate after the second injection. Local reactions have been reported as mild to moderate reactions that resolve spontaneously within 1–2 days. Systemic reactogenicity events were observed more frequently in young individuals aged 16–55 years compared to individuals over 55 years of age and occurred more frequently after the 2nd dose injections. The most common systemic events were weakness and headache (In the 16–55 age group: 51%, in the >55 age group: 39%). In the placebo group, weakness and headache were observed at a rate of 23% and 24%, respectively, following the 2nd injection. Fever above 38°C has been reported as 16% in the 16–55 age group and 11% in the >55 age group. In both the vaccine and placebo groups, the fever of 2 participants increased above 40°C. Younger participants had a higher rate of using antipyretic and other pain relievers compared to the elderly. The frequency of adverse events was reported as 27% in the vaccine group and 12% in the placebo group. Lymphadenopathy was observed in 64 participants in the vaccine arm and 6 participants in the placebo arm. Severe adverse events were reported in 4 subjects in the vaccine arm, including shoulder injury related to vaccine administration, right axillary lymphadenopathy, paroxysmal ventricular arrhythmia, and right leg paresthesia. Two BNT162b2 participants (1 due to atherosclerosis and the other due to cardiac arrest), four placebo participants (2 due to unknown causes, one hemorrhagic stroke, the other myocardial infarction) died during the trial. None of the deaths were associated with the vaccine or placebo, and no deaths from COVID-19 were reported Pfizer/Biontech Clinical Trials (2021) [online] Website https://www.pfizer.com/science/coronavirus/vaccine/rapid-progress [accessed 11 October 2020].. As per the results, the vaccine’s efficacy, administered as 30 μg in each injection with an interval of 21 days, was reported as 95%. The vaccine’s efficacy was similar in subgroups such as age, gender, ethnicity, and body mass index. Of the ten severe COVID-19 infections that occurred after the first dose of vaccination, nine occurred in the placebo arm, while one occurred in the vaccine arm. Nonsevere local and systemic side effects were common in the younger age group. Besides, it was found effective in the advanced age group, similar to those in the younger age group [10].

As a general property of mRNA vaccines, the fact that this vaccine has to be stored at very low temperatures constitutes the most important logistical problem encountered during the vaccination of large human populations. But according to our new information, the vaccine of Pfizer/Biontech can be stored 30 days at 2-8 ^0^C after taking out of deep-freeze conditions.

 It has been approved by the UK, USA, Canada, Bahrain, Saudi Arabia, and Mexico health authorities as of December 11, 2020. As of February 8, 2021, Phase IV trials have started. The results of Phase IV trials were recently reported. Between February 15, 2021, and January 8, 2023, a Phase III trial is planned for pregnant women aged 18 and over, 24-34 weeks of age Pfizer/Biontech Clinical Trials (2021) [online] Website https://clinicaltrials.gov/ct2/show/NCT04754594 [accessed 11 October 2020]..

### 2.2. Moderna 

It is an mRNA vaccine surrounded by a lipid nanoparticle (LNP). It encodes the SARS-CoV-2 spike protein. In the Phase I trial, it was observed that the more the dose administered to the participants, the higher the frequency of side effects. Also, adverse events mainly occur after the 2nd dose of the vaccine. Neutralizing antibody and RBD-binding antibody levels were studied in 34 healthy adults after 100 mcg vaccines administered 28 days apart. According to the follow-up results of the volunteers participating in the Phase I trial, the presence of neutralizing antibodies developed continues up to 90 days after the 2nd dose of the vaccine. 

No severe adverse events were observed in the vaccine arm and the placebo arm. It was reported that the vaccine has an inducing potential on the humoral immune response [11]. The vaccine’s stability was determined six months at –20 ºC, 30 days at 2–8 ºC, and 24 h at room temperature. 

In the Phase III trial conducted in the USA, mRNA-1273 (100 μg) was used as a placebo-controlled in approximately 30,420 volunteers between July 27, 2020, and October 23, 2020. While determining the vaccine’s efficacy, it was evaluated by dividing it into subgroups such as age (18–65 years old, >65 years old), gender, and ethnic origin. According to the company’s results on November 16, the vaccine’s efficacy was reported as 94.5%. All the volunteers who participated in the trial were over 18 years old. High-risk groups (> 65 years of age or those with underlying chronic diseases) constituted 42% of the trial population. The Phase III trial data, which continues with 30,420 participants in 99 centers in the USA, was published in February 2021 [12]. COVID-19 infection was detected in 2.2% of the participants in the trial (serological/virological or both). In the vaccine arm (mRNA-1273), the number of participants with symptomatic COVID-19 infection was 11, compared with 185 in the placebo group. The number of severe COVID-19 infections was 30, all of them were in the placebo group, and one resulted in death. Accordingly, protection against COVID-19 disease, including severe disease, was reported at 94.1%, and efficacy was similar in individuals aged 65 and over. The most common adverse events were headache, myalgia, fever, and pain at the injection site. These adverse events occurred more frequently in both the 1st dose and the 2nd dose vaccine groups. Also, injection site and systemic adverse events were observed more often in the younger participant subgroup (18–65 years of age). Less frequent adverse events were reported in individuals who were initially COVID-19 positive Moderna’s COVID-19 Vaccine Candidate Meets its Primary Efficacy Endpoint in the First Interim Analysis of the Phase 3 COVE Study (2020). [online] Website https://investors.modernatx.com/newsreleases/news-release-details/modernas-covid-19-vaccine-candidatemeets-its-primary-efficacy [accessed 20 November 2020]..

### 2.3. Curevac 

The Phase III trial, which started on December 14, 2020 and is expected to be completed with 36,500 participants aged 18 and over in March 2021, still continues. It is an RNA-based vaccine that will be administered at each dose of 12 µg with 28 days intervals Curevac Clinical Trials (2021) [online] Website https://clinicaltrials.gov/ct2/show/NCT04652102 [accessed 11 March 2021]..

### 2.4. Arcturus therapeutics 

ARCT-021 is a vaccine containing self-replicating (replicon) mRNA encoding the COVID-19 spike protein in a lipid nanoparticle (LNP). The placebo-controlled, double-blind Phase I trial in which 92 participants were randomized was completed in January 2021. Different doses were administered as a single injection to healthy young adults between the ages of 21–55. The efficacy, safety, and immunogenicity of these different doses were evaluated. The immunological evaluation was made by assessing the SARS-CoV-2-spike protein-specific binding antibody and neutralizing antibody titer. 

The Phase II trial, which started in January 2021 and is expected to include 600 people, is expected to be completed in June 2022 Arcturus Therapeutics Clinical Trials (2021) [online] Website https://www.clinicaltrials.gov/ct2/show/NCT04668339?term=vaccination&cond=covid&draw=1[accessed 11 March 2021]. . The trial consists of 4 different groups. These groups are as follows:

I. First dose ARCT-021 7.5 mcg-2nd dose placebo, 

II. ARCT-021 7.5mcg - ARCT-021 7.5 mcg, 

III. ARCT-021 5mcg - ARCT-021 5mcg,

IV. Placebo- Placebo. 

While evaluating the trial’s participants, local-systemic adverse events, adverse events requiring severe medical assistance, new emerging chronic diseases, biochemical and hematological changes, and SARS-CoV-2 serum neutralizing antibody titer were considered. 

### 2.5. GlaxoSmithKline 

Phase I trial of the vaccine using self-amplifying m-RNA in a lipid nanoparticle started as of February 17, 2021, and the efficacy and safety of doses of 1 µg, 3 µg, 10 µg, 30 µg in 40 volunteers aged 18–50 will be evaluated GlaxoSmithKline Clinical Trials (2021) [online] Website https://clinicaltrials.gov/ct2/show/NCT04758962[accessed 11 March 2021]..

### 2.6. Imperial College London 

Phase I trial is in progress Imperial College London Clinical Trials (2021) [online] Website https://www.isrctn.com/ISRCTN17072692[accessed 11 March 2021]..

### 2.7. Academy of Military Science (AMS)-Walvax Biotechnology 

Phase I trial is in progress Academy of Military Science (AMS)-Walvax Biotechnology (2021) [online] Website http://www.chictr.org.cn/showproj.aspx?proj=63183[accessed 11 March 2021]..

### 2.8. Providence therapeutics 

Phase I trial is in progress Providence Therapeutics Clinical Trials (2021) [online] Website https://www.clinicaltrials.gov/ct2/show/NCT04765436 [accessed 11 March 2021]..

### 2.9. Chulalongkorn University 

Phase I trial is in progress Chulalongkorn University Clinical Trials (2021) [online] Website https://clinicaltrials.gov/ct2/show/NCT04566276[accessed 11 March 2021]..

### 2.10. Moderna + National Institute of Allergy and Infectious Diseases (NIAID) 

Phase I trial is in progress Moderna + National Institute of Allergy and Infectious Diseases (NIAID) (2021) [online] Website https://www.clinicaltrials.gov/ct2/show/NCT04785144[accessed 11 March 2021]..

###  2.11. Sanofi Pasteur and Translate Bio. 

Phase I/II trial is in progress Sanofi Pasteur and Translate (2021) [online] Website https://www.clinicaltrials.gov/ct2/show/NCT04798027[accessed 11 March 2021]..

### , Ltd 2.12. Daiichi Sankyo Co., Ltd. 

Phase I/II trial is in progress Daiichi Sankyo Co., Ltd. (2021) [online] Website https://clinicaltrials.gov/ct2/show/NCT04821674 22-31[accessed 11 March 2021]..

## 3. Conclusion 

RNA-based vaccines are safe vaccines that do not have an infectious potential. They cause a potent immune response by stimulating both innate immunity and adaptive immunity with protein translation. Besides, they are stable vaccines with fast-cheap-scalable production potential. For these reasons, this technology is fundamental in COVID-19 and other infectious diseases. In the following days, we will see and use RNA-based vaccines more effectively. 

## References

[ref1] Sencan I Kuzi S. The global threat of COVID-19 and evacuation of the citizens of different countries Turkish Journal Medical Sciences 2020 April 21 534 543 10.3906/sag-2004-21 PMC7195976 32283903

[ref2] Goshua G Pine AB Meizlish ML, Chang CH, Zhang H et al Lancet Haematology 10 e575 e582 10.1016/S2352-3026(20)30216-7 PMC7326446 32619411

[ref3] Xie Y You Q Wu C Cao S Impact of Cardiovascular Disease on Clinical Characteristics and Outcomes of Coronavirus Disease 2019 (COVID-19) 22 1277 1283 10.1253/circj.CJ-20-0348 32536672

[ref4] Al-Samkari H Carlson JCT Fogerty AE -19 and coagulation: bleeding and thrombotic manifestations of SARS-CoV-2 infection 2020 July 23 489 500 10.1182/blood.2020006520 PMC7378457 32492712

[ref5] Hu K Patel J Swiston C Patel BC 2021 May 19 Ophthalmic Manifestations of Coronavirus (COVID-19) 32310553

[ref6] Ferrarese Silani V Galimberti A 2020 An Italian multicenter retrospective-prospective observational study on neurological manifestations of COVID-19 41 1355 1359 10.1007/s10072-020-04450-1 PMC7235538 32430621

[ref7] Pardi N Hogan MJ Porter FW Weissman D 2018 a new vaccinology area Nature Reviews Drug Discovery 17 261 279 29326426 10.1038/nrd.2017.243 PMC5906799

[ref8] Mulligan MJ Lyke KE Kitchin N Absalon J Gurtman A Nature 586 589 593 32785213 10.1038/s41586-020-2639-4

[ref9] Walsh EE Frenck RW Jr. Falsey AR Kitchin N Absalon J 2020 Safety and Immunogenicity of Two RNA-Based Covid-Vaccine Candidates, New England Journal of Medicine 383 2439 2450 10.1056/NEJMoa2027906 PMC7583697 33053279

[ref10] Polack FP Thomas SJ Kitchin N Absalon J Gurtman A Safety and Efficacy of the BNT162b2 mRNA Covid- Vaccine, New England Journal of Medicine 31 2603 2615 10.1056/NEJMoa2034577 PMC7745181 33301246

[ref11] Widge AT Rouphel NG Jackson LA Anderson AJ Roberts PJ Durability of responses after SARS-CoV-2 mRNA- vaccination. New England Journal of Medicine 7 80 82 10.1056/NEJMc2032195 PMC7727324 33270381

[ref12] Baden RL El Sahly Essink B Kotloff K Efficacy and Safety of the mRNA-1273 SARS-CoV-2 Vaccine, New England Journal of Medicine 4 403 416 10.1056/NEJMoa2035389 PMC7787219 33378609

